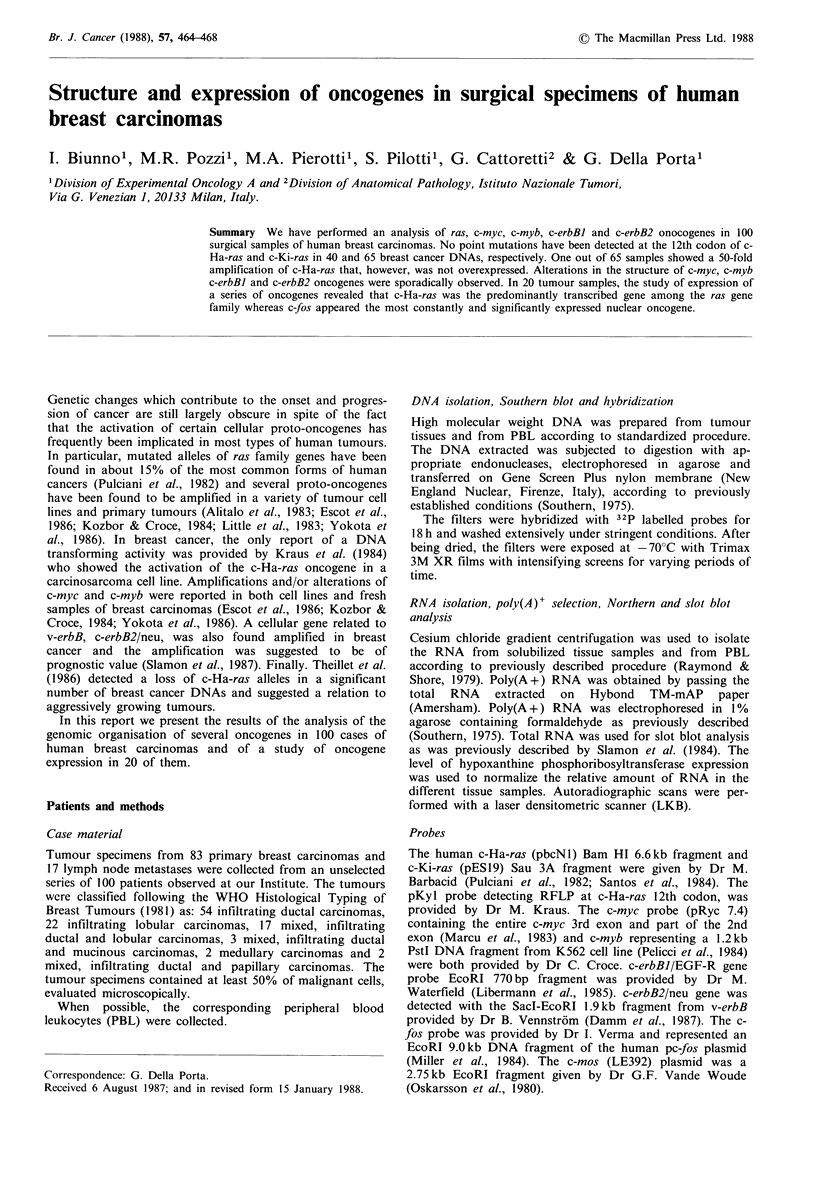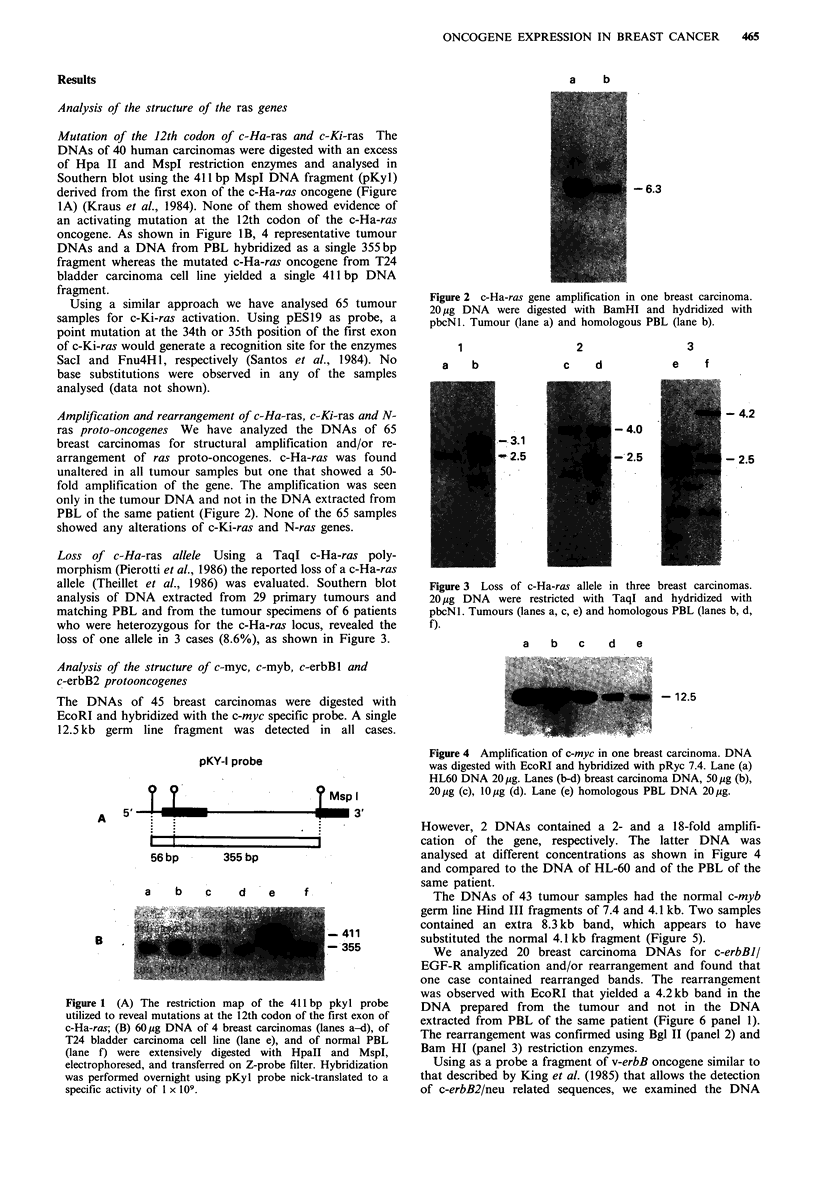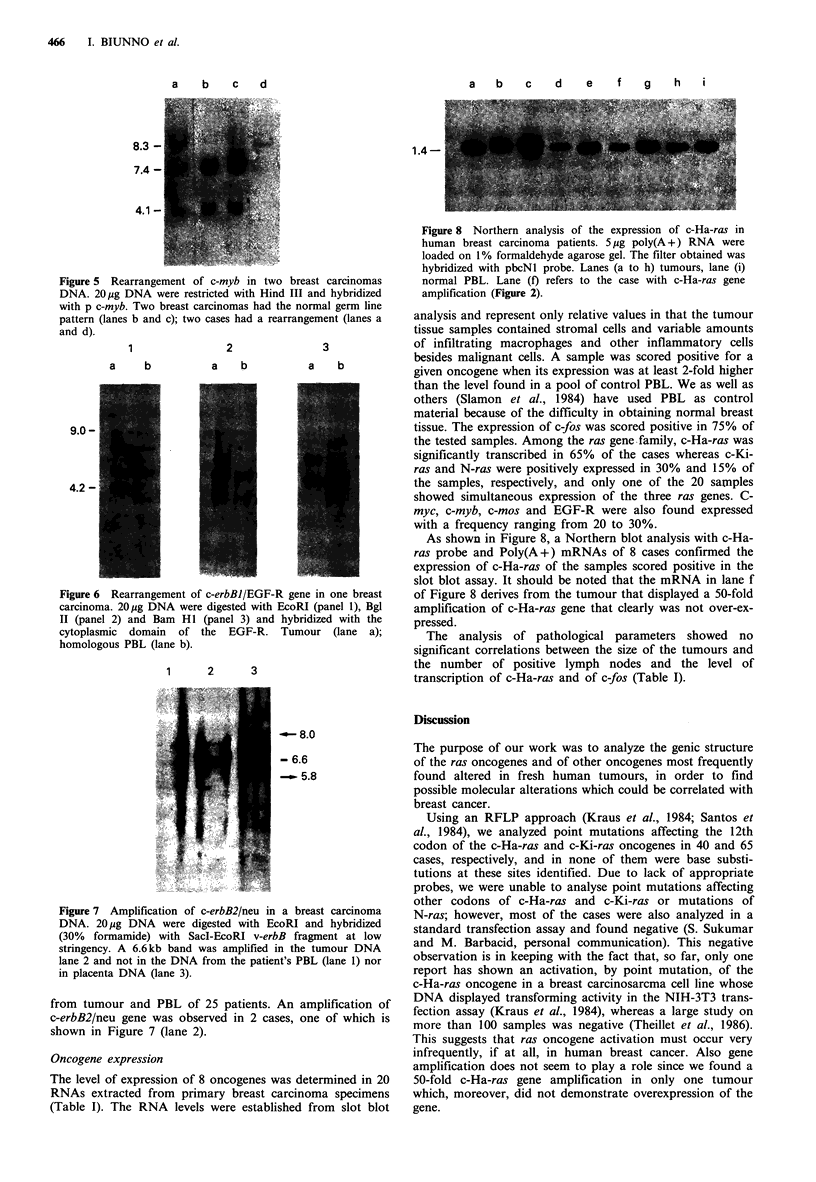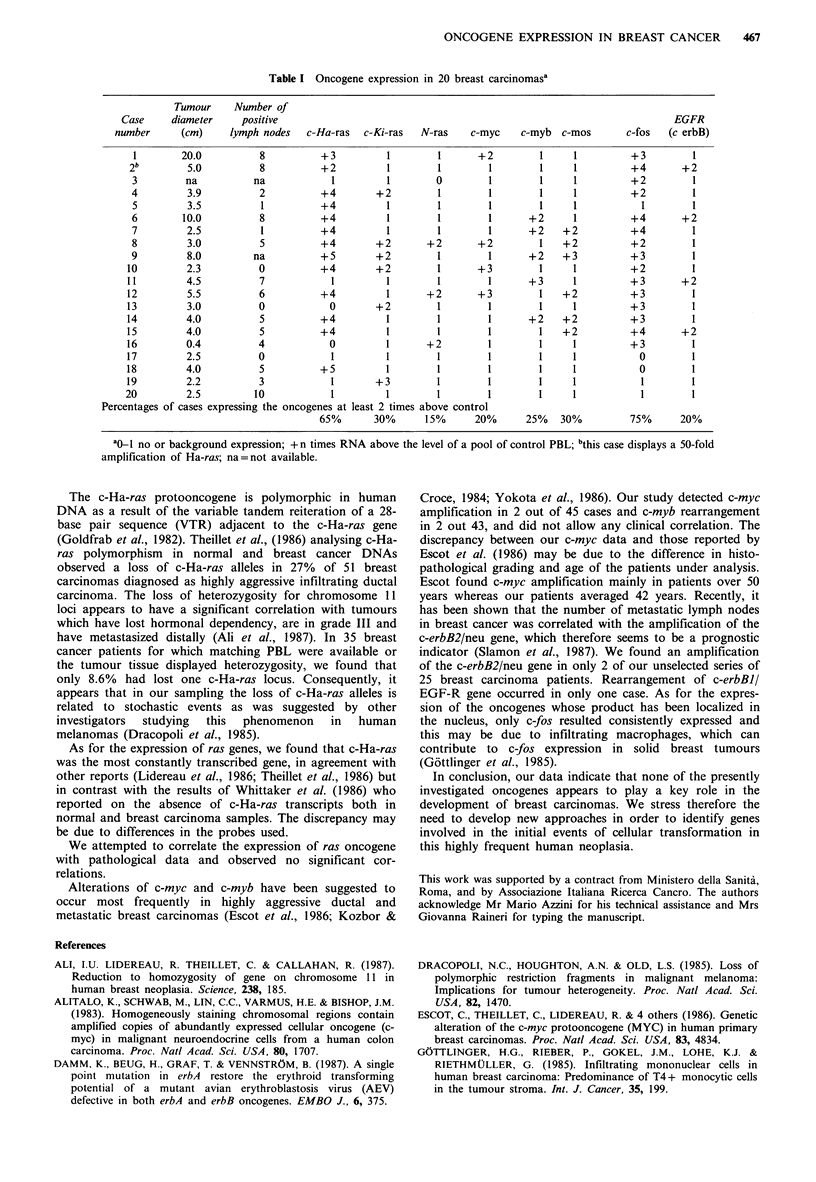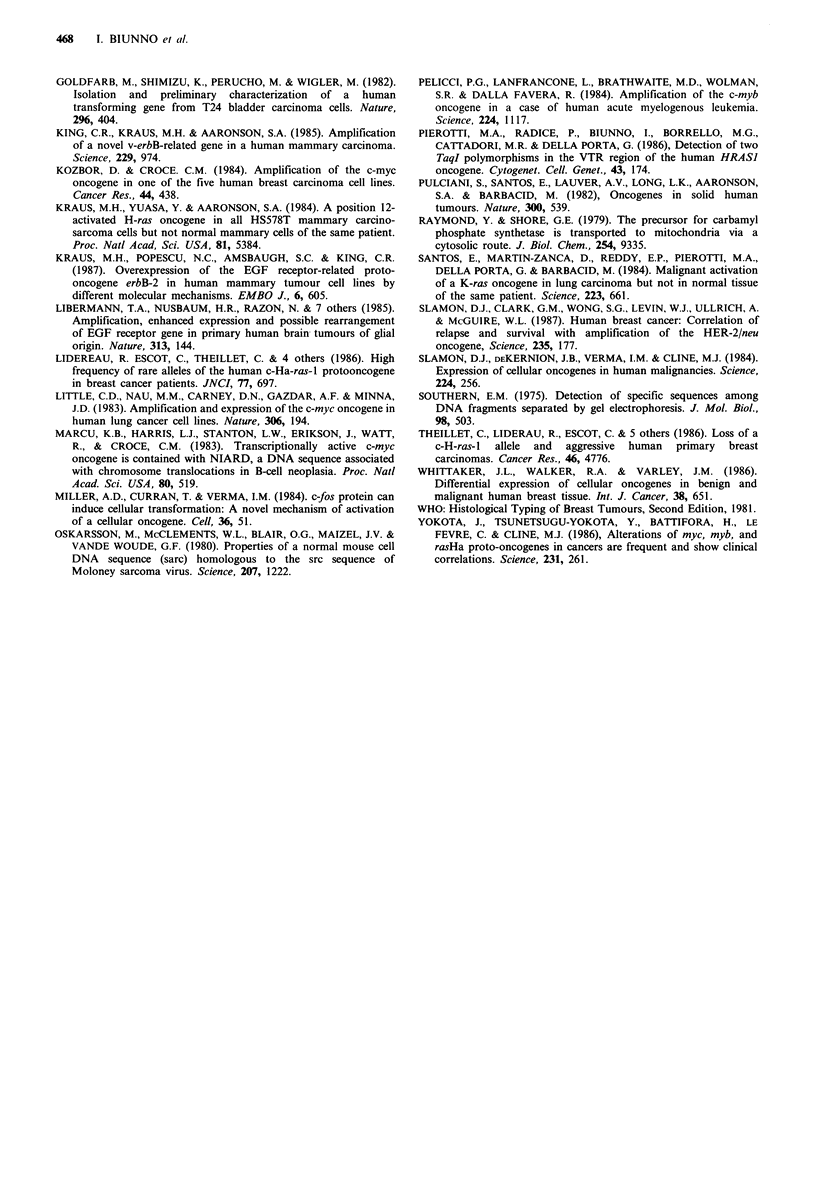# Structure and expression of oncogenes in surgical specimens of human breast carcinomas.

**DOI:** 10.1038/bjc.1988.108

**Published:** 1988-05

**Authors:** I. Biunno, M. R. Pozzi, M. A. Pierotti, S. Pilotti, G. Cattoretti, G. Della Porta

**Affiliations:** Division of Experimental Oncology A, Istituto Nazionale Tumori, Milan, Italy.

## Abstract

**Images:**


Br. J. Cancer (1988), 57, 464–468 © The Macmillan Press Ltd. 1988

### Structure and expression of oncogenes in surgical specimens of human breast carcinomas

I. Biunno[1], M.R. Pozzi[1], M.A. Pierotti[1], S. Pilotti[1], G. Cattoretti[2] & G. Della Porta[1]

[1]*Division of Experimental Oncology A and* [2]*Division of Anatomical Pathology, Istituto Nazionale Tumori, Via G. Venezian 1, 20133 Milan, Italy.*

**Summary** We have performed an analysis of *ras*, c-*myc*, c-*myb*, c-*erbB1* and c-*erbB2* onocogenes in 100 surgical samples of human breast carcinomas. No point mutations have been detected at the 12th codon of c-Ha-*ras* and c-Ki-*ras* in 40 and 65 breast cancer DNAs, respectively. One out of 65 samples showed a 50-fold amplification of c-Ha-*ras* that, however, was not overexpressed. Alterations in the structure of c-*myc*, c-*myb* c-*erbB1* and c-*erbB2* oncogenes were sporadically observed. In 20 tumour samples, the study of expression of a series of oncogenes revealed that c-Ha-*ras* was the predominantly transcribed gene among the *ras* gene family whereas c-*fos* appeared the most constantly and significantly expressed nuclear oncogene.

Genetic changes which contribute to the onset and progression of cancer are still largely obscure in spite of the fact that the activation of certain cellular proto-oncogenes has frequently been implicated in most types of human tumours. In particular, mutated alleles of *ras* family genes have been found in about 15% of the most common forms of human cancers (Pulciani *et al.*, 1982) and several proto-oncogenes have been found to be amplified in a variety of tumour cell lines and primary tumours (Alitalo *et al.*, 1983; Escot *et al.*, 1986; Kozbor & Croce, 1984; Little *et al.*, 1983; Yokota *et al.*, 1986). In breast cancer, the only report of a DNA transforming activity was provided by Kraus *et al.* (1984) who showed the activation of the c-Ha-*ras* oncogene in a carcinosarcoma cell line. Amplifications and/or alterations of c-*myc* and c-*myb* were reported in both cell lines and fresh samples of breast carcinomas (Escot *et al.*, 1986; Kozbor & Croce, 1984; Yokota *et al.*, 1986). A cellular gene related to v-*erbB*, c-*erbB2*/neu, was also found amplified in breast cancer and the amplification was suggested to be of prognostic value (Slamon *et al.*, 1987). Finally. Theillet *et al.* (1986) detected a loss of c-Ha-*ras* alleles in a significant number of breast cancer DNAs and suggested a relation to aggressively growing tumours.

In this report we present the results of the analysis of the genomic organisation of several oncogenes in 100 cases of human breast carcinomas and of a study of oncogene expression in 20 of them.

### Patients and methods

### *Case material*

Tumour specimens from 83 primary breast carcinomas and 17 lymph node metastases were collected from an unselected series of 100 patients observed at our Institute. The tumours were classified following the WHO Histological Typing of Breast Tumours (1981) as: 54 infiltrating ductal carcinomas, 22 infiltrating lobular carcinomas, 17 mixed, infiltrating ductal and lobular carcinomas, 3 mixed, infiltrating ductal and mucinous carcinomas, 2 medullary carcinomas and 2 mixed, infiltrating ductal and papillary carcinomas. The tumour specimens contained at least 50% of malignant cells, evaluated microscopically.

When possible, the corresponding peripheral blood leukocytes (PBL) were collected.

Correspondence: G. Della Porta.
Received 6 August 1987; and in revised form 15 January 1988.

### *DNA isolation, Southern blot and hybridization*

High molecular weight DNA was prepared from tumour tissues and from PBL according to standardized procedure. The DNA extracted was subjected to digestion with appropriate endonucleases, electrophoresed in agarose and transferred on Gene Screen Plus nylon membrane (New England Nuclear, Firenze, Italy), according to previously established conditions (Southern, 1975).

The filters were hybridized with $^{32}P$ labelled probes for 18 h and washed extensively under stringent conditions. After being dried, the filters were exposed at $-70°C$ with Trimax 3M XR films with intensifying screens for varying periods of time.

### *RNA isolation, poly(A)$^+$ selection, Northern and slot blot analysis*

Cesium chloride gradient centrifugation was used to isolate the RNA from solubilized tissue samples and from PBL according to previously described procedure (Raymond & Shore, 1979). Poly(A+) RNA was obtained by passing the total RNA extracted on Hybond TM-mAP paper (Amersham). Poly(A+) RNA was electrophoresed in 1% agarose containing formaldehyde as previously described (Southern, 1975). Total RNA was used for slot blot analysis as was previously described by Slamon *et al.* (1984). The level of hypoxanthine phosphoribosyltransferase expression was used to normalize the relative amount of RNA in the different tissue samples. Autoradiographic scans were performed with a laser densitometric scanner (LKB).

### *Probes*

The human c-Ha-*ras* (pbcN1) Bam HI 6.6 kb fragment and c-Ki-*ras* (pES19) Sau 3A fragment were given by Dr M. Barbacid (Pulciani *et al.*, 1982; Santos *et al.*, 1984). The pKyl probe detecting RFLP at c-Ha-*ras* 12th codon, was provided by Dr M. Kraus. The c-*myc* probe (pRyc 7.4) containing the entire c-*myc* 3rd exon and part of the 2nd exon (Marcu *et al.*, 1983) and c-*myb* representing a 1.2 kb PstI DNA fragment from K562 cell line (Pelicci *et al.*, 1984) were both provided by Dr C. Croce. c-*erbB1*/EGF-R gene probe EcoRI 770 bp fragment was provided by Dr M. Waterfield (Libermann *et al.*, 1985). c-*erbB2*/neu gene was detected with the SacI-EcoRI 1.9 kb fragment from v-*erbB* provided by Dr B. Vennström (Damm *et al.*, 1987). The c-*fos* probe was provided by Dr I. Verma and represented an EcoRI 9.0 kb DNA fragment of the human pc-*fos* plasmid (Miller *et al.*, 1984). The c-*mos* (LE392) plasmid was a 2.75 kb EcoRI fragment given by Dr G.F. Vande Woude (Oskarsson *et al.*, 1980).

### Results

*Analysis of the structure of the* ras *genes*

*Mutation of the 12th codon of c-Ha-*ras *and c-Ki-*ras The DNAs of 40 human carcinomas were digested with an excess of Hpa II and MspI restriction enzymes and analysed in Southern blot using the 411 bp MspI DNA fragment (pKy1) derived from the first exon of the c-Ha-*ras* oncogene (Figure 1A) (Kraus *et al.*, 1984). None of them showed evidence of an activating mutation at the 12th codon of the c-Ha-*ras* oncogene. As shown in Figure 1B, 4 representative tumour DNAs and a DNA from PBL hybridized as a single 355 bp fragment whereas the mutated c-Ha-*ras* oncogene from T24 bladder carcinoma cell line yielded a single 411 bp DNA fragment.

Using a similar approach we have analysed 65 tumour samples for c-Ki-*ras* activation. Using pES19 as probe, a point mutation at the 34th or 35th position of the first exon of c-Ki-*ras* would generate a recognition site for the enzymes SacI and Fnu4H1, respectively (Santos *et al.*, 1984). No base substitutions were observed in any of the samples analysed (data not shown).

*Amplification and rearrangement of c-Ha-*ras*, c-Ki-*ras *and N-*ras *proto-oncogenes* We have analyzed the DNAs of 65 breast carcinomas for structural amplification and/or rearrangement of *ras* proto-oncogenes. c-Ha-*ras* was found unaltered in all tumour samples but one that showed a 50-fold amplification of the gene. The amplification was seen only in the tumour DNA and not in the DNA extracted from PBL of the same patient (Figure 2). None of the 65 samples showed any alterations of c-Ki-*ras* and N-*ras* genes.

*Loss of c-Ha-*ras *allele* Using a TaqI c-Ha-*ras* polymorphism (Pierotti *et al.*, 1986) the reported loss of a c-Ha-*ras* allele (Theillet *et al.*, 1986) was evaluated. Southern blot analysis of DNA extracted from 29 primary tumours and matching PBL and from the tumour specimens of 6 patients who were heterozygous for the c-Ha-*ras* locus, revealed the loss of one allele in 3 cases (8.6%), as shown in Figure 3.

*Analysis of the structure of* c-myc, c-myb, c-erbB1 *and* c-erbB2 *protooncogenes*

The DNAs of 45 breast carcinomas were digested with EcoRI and hybridized with the c-*myc* specific probe. A single 12.5 kb germ line fragment was detected in all cases.

**Figure 1** (A) The restriction map of the 411 bp pky1 probe utilized to reveal mutations at the 12th codon of the first exon of c-Ha-*ras*; (B) 60 μg DNA of 4 breast carcinomas (lanes a–d), of T24 bladder carcinoma cell line (lane e), and of normal PBL (lane f) were extensively digested with HpaII and MspI, electrophoresed, and transferred on Z-probe filter. Hybridization was performed overnight using pKy1 probe nick-translated to a specific activity of $1 \times 10^9$.

**Figure 2** c-Ha-*ras* gene amplification in one breast carcinoma. 20 μg DNA were digested with BamHI and hydridized with pbcN1. Tumour (lane a) and homologous PBL (lane b).

**Figure 3** Loss of c-Ha-*ras* allele in three breast carcinomas. 20 μg DNA were restricted with TaqI and hydridized with pbcN1. Tumours (lanes a, c, e) and homologous PBL (lanes b, d, f).

**Figure 4** Amplification of c-*myc* in one breast carcinoma. DNA was digested with EcoRI and hybridized with pRyc 7.4. Lane (a) HL60 DNA 20 μg. Lanes (b-d) breast carcinoma DNA, 50 μg (b), 20 μg (c), 10 μg (d). Lane (e) homologous PBL DNA 20 μg.

However, 2 DNAs contained a 2- and a 18-fold amplification of the gene, respectively. The latter DNA was analysed at different concentrations as shown in Figure 4 and compared to the DNA of HL-60 and of the PBL of the same patient.

The DNAs of 43 tumour samples had the normal c-*myb* germ line Hind III fragments of 7.4 and 4.1 kb. Two samples contained an extra 8.3 kb band, which appears to have substituted the normal 4.1 kb fragment (Figure 5).

We analyzed 20 breast carcinoma DNAs for c-*erbB1*/EGF-R amplification and/or rearrangement and found that one case contained rearranged bands. The rearrangement was observed with EcoRI that yielded a 4.2 kb band in the DNA prepared from the tumour and not in the DNA extracted from PBL of the same patient (Figure 6 panel 1). The rearrangement was confirmed using Bgl II (panel 2) and Bam HI (panel 3) restriction enzymes.

Using as a probe a fragment of v-*erbB* oncogene similar to that described by King *et al.* (1985) that allows the detection of c-*erbB2*/neu related sequences, we examined the DNA

**Figure 5** Rearrangement of c-*myb* in two breast carcinomas DNA. 20 μg DNA were restricted with Hind III and hybridized with p c-*myb*. Two breast carcinomas had the normal germ line pattern (lanes b and c); two cases had a rearrangement (lanes a and d).

**Figure 6** Rearrangement of c-*erbB1*/EGF-R gene in one breast carcinoma. 20 μg DNA were digested with EcoRI (panel 1), Bgl II (panel 2) and Bam H1 (panel 3) and hybridized with the cytoplasmic domain of the EGF-R. Tumour (lane a); homologous PBL (lane b).

**Figure 7** Amplification of c-*erbB2*/neu in a breast carcinoma DNA. 20 μg DNA were digested with EcoRI and hybridized (30% formamide) with SacI-EcoRI v-*erbB* fragment at low stringency. A 6.6 kb band was amplified in the tumour DNA lane 2 and not in the DNA from the patient's PBL (lane 1) nor in placenta DNA (lane 3).

from tumour and PBL of 25 patients. An amplification of c-*erbB2*/neu gene was observed in 2 cases, one of which is shown in Figure 7 (lane 2).

*Oncogene expression*

The level of expression of 8 oncogenes was determined in 20 RNAs extracted from primary breast carcinoma specimens (Table I). The RNA levels were established from slot blot

**Figure 8** Northern analysis of the expression of c-Ha-*ras* in human breast carcinoma patients. 5 μg poly(A+) RNA were loaded on 1% formaldehyde agarose gel. The filter obtained was hybridized with pbcN1 probe. Lanes (a to h) tumours, lane (i) normal PBL. Lane (f) refers to the case with c-Ha-*ras* gene amplification (**Figure 2**).

analysis and represent only relative values in that the tumour tissue samples contained stromal cells and variable amounts of infiltrating macrophages and other inflammatory cells besides malignant cells. A sample was scored positive for a given oncogene when its expression was at least 2-fold higher than the level found in a pool of control PBL. We as well as others (Slamon *et al.*, 1984) have used PBL as control material because of the difficulty in obtaining normal breast tissue. The expression of c-*fos* was scored positive in 75% of the tested samples. Among the *ras* gene family, c-Ha-*ras* was significantly transcribed in 65% of the cases whereas c-Ki-*ras* and N-*ras* were positively expressed in 30% and 15% of the samples, respectively, and only one of the 20 samples showed simultaneous expression of the three *ras* genes. C-*myc*, c-*myb*, c-*mos* and EGF-R were also found expressed with a frequency ranging from 20 to 30%.

As shown in Figure 8, a Northern blot analysis with c-Ha-*ras* probe and Poly(A+) mRNAs of 8 cases confirmed the expression of c-Ha-*ras* of the samples scored positive in the slot blot assay. It should be noted that the mRNA in lane f of Figure 8 derives from the tumour that displayed a 50-fold amplification of c-Ha-*ras* gene that clearly was not over-expressed.

The analysis of pathological parameters showed no significant correlations between the size of the tumours and the number of positive lymph nodes and the level of transcription of c-Ha-*ras* and of c-*fos* (Table I).

### Discussion

The purpose of our work was to analyze the genic structure of the *ras* oncogenes and of other oncogenes most frequently found altered in fresh human tumours, in order to find possible molecular alterations which could be correlated with breast cancer.

Using an RFLP approach (Kraus *et al.*, 1984; Santos *et al.*, 1984), we analyzed point mutations affecting the 12th codon of the c-Ha-*ras* and c-Ki-*ras* oncogenes in 40 and 65 cases, respectively, and in none of them were base substitutions at these sites identified. Due to lack of appropriate probes, we were unable to analyse point mutations affecting other codons of c-Ha-*ras* and c-Ki-*ras* or mutations of N-*ras*; however, most of the cases were also analyzed in a standard transfection assay and found negative (S. Sukumar and M. Barbacid, personal communication). This negative observation is in keeping with the fact that, so far, only one report has shown an activation, by point mutation, of the c-Ha-*ras* oncogene in a breast carcinosarcma cell line whose DNA displayed transforming activity in the NIH-3T3 transfection assay (Kraus *et al.*, 1984), whereas a large study on more than 100 samples was negative (Theillet *et al.*, 1986). This suggests that *ras* oncogene activation must occur very infrequently, if at all, in human breast cancer. Also gene amplification does not seem to play a role since we found a 50-fold c-Ha-*ras* gene amplification in only one tumour which, moreover, did not demonstrate overexpression of the gene.

**Table I** Oncogene expression in 20 breast carcinomas[a]

| Case number | Tumour diameter (cm) | Number of positive lymph nodes | c-*Ha*-ras | c-*Ki*-ras | *N*-ras | c-myc | c-myb | c-mos | c-fos | EGFR (c erbB) |
|---|---|---|---|---|---|---|---|---|---|---|
| 1 | 20.0 | 8 | +3 | 1 | 1 | +2 | 1 | 1 | +3 | 1 |
| 2[b] | 5.0 | 8 | +2 | 1 | 1 | 1 | 1 | 1 | +4 | +2 |
| 3 | na | na | 1 | 1 | 0 | 1 | 1 | 1 | +2 | 1 |
| 4 | 3.9 | 2 | +4 | +2 | 1 | 1 | 1 | 1 | +2 | 1 |
| 5 | 3.5 | 1 | +4 | 1 | 1 | 1 | 1 | 1 | 1 | 1 |
| 6 | 10.0 | 8 | +4 | 1 | 1 | 1 | +2 | 1 | +4 | +2 |
| 7 | 2.5 | 1 | +4 | 1 | 1 | 1 | +2 | +2 | +4 | 1 |
| 8 | 3.0 | 5 | +4 | +2 | +2 | +2 | 1 | +2 | +2 | 1 |
| 9 | 8.0 | na | +5 | +2 | 1 | 1 | +2 | +3 | +3 | 1 |
| 10 | 2.3 | 0 | +4 | +2 | 1 | +3 | 1 | 1 | +2 | 1 |
| 11 | 4.5 | 7 | 1 | 1 | 1 | 1 | +3 | 1 | +3 | +2 |
| 12 | 5.5 | 6 | +4 | 1 | +2 | +3 | 1 | +2 | +3 | 1 |
| 13 | 3.0 | 0 | 0 | +2 | 1 | 1 | 1 | 1 | +3 | 1 |
| 14 | 4.0 | 5 | +4 | 1 | 1 | 1 | +2 | +2 | +3 | 1 |
| 15 | 4.0 | 5 | +4 | 1 | 1 | 1 | 1 | +2 | +4 | +2 |
| 16 | 0.4 | 4 | 0 | 1 | +2 | 1 | 1 | 1 | +3 | 1 |
| 17 | 2.5 | 0 | 1 | 1 | 1 | 1 | 1 | 1 | 0 | 1 |
| 18 | 4.0 | 5 | +5 | 1 | 1 | 1 | 1 | 1 | 0 | 1 |
| 19 | 2.2 | 3 | 1 | +3 | 1 | 1 | 1 | 1 | 1 | 1 |
| 20 | 2.5 | 10 | 1 | 1 | 1 | 1 | 1 | 1 | 1 | 1 |
| Percentages of cases expressing the oncogenes at least 2 times above control | | | 65% | 30% | 15% | 20% | 25% | 30% | 75% | 20% |

[a]0–1 no or background expression; +n times RNA above the level of a pool of control PBL; [b]this case displays a 50-fold amplification of Ha-*ras*; na = not available.

The c-Ha-*ras* protooncogene is polymorphic in human DNA as a result of the variable tandem reiteration of a 28-base pair sequence (VTR) adjacent to the c-Ha-*ras* gene (Goldfrab *et al.*, 1982). Theillet *et al.*, (1986) analysing c-Ha-*ras* polymorphism in normal and breast cancer DNAs observed a loss of c-Ha-*ras* alleles in 27% of 51 breast carcinomas diagnosed as highly aggressive infiltrating ductal carcinoma. The loss of heterozygosity for chromosome 11 loci appears to have a significant correlation with tumours which have lost hormonal dependency, are in grade III and have metastasized distally (Ali *et al.*, 1987). In 35 breast cancer patients for which matching PBL were available or the tumour tissue displayed heterozygosity, we found that only 8.6% had lost one c-Ha-*ras* locus. Consequently, it appears that in our sampling the loss of c-Ha-*ras* alleles is related to stochastic events as was suggested by other investigators studying this phenomenon in human melanomas (Dracopoli *et al.*, 1985).

As for the expression of *ras* genes, we found that c-Ha-*ras* was the most constantly transcribed gene, in agreement with other reports (Lidereau *et al.*, 1986; Theillet *et al.*, 1986) but in contrast with the results of Whittaker *et al.* (1986) who reported on the absence of c-Ha-*ras* transcripts both in normal and breast carcinoma samples. The discrepancy may be due to differences in the probes used.

We attempted to correlate the expression of *ras* oncogene with pathological data and observed no significant correlations.

Alterations of c-*myc* and c-*myb* have been suggested to occur most frequently in highly aggressive ductal and metastatic breast carcinomas (Escot *et al.*, 1986; Kozbor & Croce, 1984; Yokota *et al.*, 1986). Our study detected c-*myc* amplification in 2 out of 45 cases and c-*myb* rearrangement in 2 out 43, and did not allow any clinical correlation. The discrepancy between our c-*myc* data and those reported by Escot *et al.* (1986) may be due to the difference in histopathological grading and age of the patients under analysis. Escot found c-*myc* amplification mainly in patients over 50 years whereas our patients averaged 42 years. Recently, it has been shown that the number of metastatic lymph nodes in breast cancer was correlated with the amplification of the c-*erbB2*/neu gene, which therefore seems to be a prognostic indicator (Slamon *et al.*, 1987). We found an amplification of the c-*erbB2*/neu gene in only 2 of our unselected series of 25 breast carcinoma patients. Rearrangement of c-*erbB1*/EGF-R gene occurred in only one case. As for the expression of the oncogenes whose product has been localized in the nucleus, only c-*fos* resulted consistently expressed and this may be due to infiltrating macrophages, which can contribute to c-*fos* expression in solid breast tumours (Göttlinger *et al.*, 1985).

In conclusion, our data indicate that none of the presently investigated oncogenes appears to play a key role in the development of breast carcinomas. We stress therefore the need to develop new approaches in order to identify genes involved in the initial events of cellular transformation in this highly frequent human neoplasia.

This work was supported by a contract from Ministero della Sanità, Roma, and by Associazione Italiana Ricerca Cancro. The authors acknowledge Mr Mario Azzini for his technical assistance and Mrs Giovanna Raineri for typing the manuscript.

**References**

ALI, I.U. LIDEREAU, R. THEILLET, C. & CALLAHAN, R. (1987). Reduction to homozygosity of gene on chromosome 11 in human breast neoplasia. *Science*, **238**, 185.

ALITALO, K., SCHWAB, M., LIN, C.C., VARMUS, H.E. & BISHOP, J.M. (1983). Homogeneously staining chromosomal regions contain amplified copies of abundantly expressed cellular oncogene (c-myc) in malignant neuroendocrine cells from a human colon carcinoma. *Proc. Natl Acad. Sci. USA*, **80**, 1707.

DAMM, K., BEUG, H., GRAF, T. & VENNSTRÖM, B. (1987). A single point mutation in *erbA* restore the erythroid transforming potential of a mutant avian erythroblastosis virus (AEV) defective in both *erbA* and *erbB* oncogenes. *EMBO J.*, **6**, 375.

DRACOPOLI, N.C., HOUGHTON, A.N. & OLD, L.S. (1985). Loss of polymorphic restriction fragments in malignant melanoma: Implications for tumour heterogeneity. *Proc. Natl Acad. Sci. USA*, **82**, 1470.

ESCOT, C., THEILLET, C., LIDEREAU, R. & 4 others (1986). Genetic alteration of the c-*myc* protooncogene (MYC) in human primary breast carcinomas. *Proc. Natl Acad. Sci. USA*, **83**, 4834.

GÖTTLINGER, H.G., RIEBER, P., GOKEL, J.M., LOHE, K.J. & RIETHMÜLLER, G. (1985). Infiltrating mononuclear cells in human breast carcinoma: Predominance of T4+ monocytic cells in the tumour stroma. *Int. J. Cancer*, **35**, 199.

GOLDFARB, M., SHIMIZU, K., PERUCHO, M. & WIGLER, M. (1982). Isolation and preliminary characterization of a human transforming gene from T24 bladder carcinoma cells. *Nature*, **296**, 404.

KING, C.R., KRAUS, M.H. & AARONSON, S.A. (1985). Amplification of a novel v-*erb*B-related gene in a human mammary carcinoma. *Science*, **229**, 974.

KOZBOR, D. & CROCE. C.M. (1984). Amplification of the c-myc oncogene in one of the five human breast carcinoma cell lines. *Cancer Res.*, **44**, 438.

KRAUS, M.H., YUASA, Y. & AARONSON, S.A. (1984). A position 12-activated H-*ras* oncogene in all HS578T mammary carcinosarcoma cells but not normal mammary cells of the same patient. *Proc. Natl Acad, Sci. USA*, **81**, 5384.

KRAUS, M.H., POPESCU, N.C., AMSBAUGH, S.C. & KING, C.R. (1987). Overexpression of the EGF receptor-related proto-oncogene *erb*B-2 in human mammary tumour cell lines by different molecular mechanisms. *EMBO J.*, **6**, 605.

LIBERMANN, T.A., NUSBAUM, H.R., RAZON, N. & 7 others (1985). Amplification, enhanced expression and possible rearrangement of EGF receptor gene in primary human brain tumours of glial origin. *Nature*, **313**, 144.

LIDEREAU, R. ESCOT, C., THEILLET, C. & 4 others (1986). High frequency of rare alleles of the human c-Ha-*ras*-1 protooncogene in breast cancer patients. *JNCI*, **77**, 697.

LITTLE, C.D., NAU, M.M., CARNEY, D.N., GAZDAR, A.F. & MINNA, J.D. (1983). Amplification and expression of the c-*myc* oncogene in human lung cancer cell lines. *Nature*, **306**, 194.

MARCU, K.B., HARRIS, L.J., STANTON, L.W., ERIKSON, J., WATT, R., & CROCE, C.M. (1983). Transcriptionally active c-*myc* oncogene is contained with NIARD, a DNA sequence associated with chromosome translocations in B-cell neoplasia. *Proc. Natl Acad. Sci. USA*, **80**, 519.

MILLER, A.D., CURRAN, T. & VERMA, I.M. (1984). c-*fos* protein can induce cellular transformation: A novel mechanism of activation of a cellular oncogene. *Cell*, **36**, 51.

OSKARSSON, M., MCCLEMENTS, W.L., BLAIR, O.G., MAIZEL, J.V. & VANDE WOUDE, G.F. (1980). Properties of a normal mouse cell DNA sequence (sarc) homologous to the src sequence of Moloney sarcoma virus. *Science*, **207**, 1222.

PELICCI, P.G., LANFRANCONE, L., BRATHWAITE, M.D., WOLMAN, S.R. & DALLA FAVERA, R. (1984). Amplification of the c-*myb* oncogene in a case of human acute myelogenous leukemia. *Science*, **224**, 1117.

PIEROTTI, M.A., RADICE, P., BIUNNO, I., BORRELLO, M.G., CATTADORI, M.R. & DELLA PORTA, G. (1986), Detection of two *TaqI* polymorphisms in the VTR region of the human *HRAS1* oncogene. *Cytogenet. Cell. Genet.*, **43**, 174.

PULCIANI, S., SANTOS, E., LAUVER, A.V., LONG, L.K., AARONSON, S.A. & BARBACID, M. (1982), Oncogenes in solid human tumours. *Nature*, **300**, 539.

RAYMOND, Y. & SHORE, G.E. (1979). The precursor for carbamyl phosphate synthetase is transported to mitochondria via a cytosolic route. *J. Biol. Chem.*, **254**, 9335.

SANTOS, E., MARTIN-ZANCA, D., REDDY, E.P., PIEROTTI, M.A., DELLA PORTA, G. & BARBACID, M. (1984). Malignant activation of a K-*ras* oncogene in lung carcinoma but not in normal tissue of the same patient. *Science*, **223**, 661.

SLAMON, D.J., CLARK, G.M., WONG, S.G., LEVIN, W.J., ULLRICH, A. & MCGUIRE, W.L. (1987). Human breast cancer: Correlation of relapse and survival with amplification of the HER-2/*neu* oncogene, *Science*, **235**, 177.

SLAMON, D.J., DEKERNION, J.B., VERMA, I.M. & CLINE, M.J. (1984). Expression of cellular oncogenes in human malignancies. *Science*, **224**, 256.

SOUTHERN, E.M. (1975). Detection of specific sequences among DNA fragments separated by gel electrophoresis. *J. Mol. Biol.*, **98**, 503.

THEILLET, C., LIDERAU, R., ESCOT, C. & 5 others (1986). Loss of a c-H-*ras*-1 allele and aggressive human primary breast carcinomas. *Cancer Res.*, **46**, 4776.

WHITTAKER, J.L., WALKER, R.A. & VARLEY, J.M. (1986). Differential expression of cellular oncogenes in benign and malignant human breast tissue. *Int. J. Cancer*, **38**, 651.

WHO: Histological Typing of Breast Tumours, Second Edition, 1981.

YOKOTA, J., TSUNETSUGU-YOKOTA, Y., BATTIFORA, H., LE FEVRE, C. & CLINE, M.J. (1986), Alterations of *myc*, *myb*, and *ras*Ha proto-oncogenes in cancers are frequent and show clinical correlations. *Science*, **231**, 261.